# Stoddard Bros.

**Published:** 1890-08

**Authors:** 


Stoddard Bros., of No. 84 Seneca street, have recently opened an instrument department in connection with their drug store. This enterprising firm deserves the thanks and support of the profession in their new enterprise. They will handle the instruments of the leading makers and will be glad to obtain any instrument not in stock on the shortest possible time. Mr. George B. McCullough has been secured as the manager of this department. Mr. McCullough has had a large experience in the instrument business, and is besides a most courteous gentleman. We have no doubt but in his hands this department will be made not only successful for the proprietors, but attractive to the profession, and that those who heretofore have frequently been compelled to send out of town for their instruments, will find it more convenient and just as satisfactory to purchase through this firm.

